# Comparison of Surgical Site Infection After Skin Closure by Prolene or Staples in Bilateral Simultaneous Knee Arthroplasty Patients: A Parallel Design Randomized Controlled Trial Protocol

**DOI:** 10.29337/ijsp.153

**Published:** 2021-08-06

**Authors:** Obada Hasan, Ahsun Jiwani, Laraib Mazhar, Dilshad Begum, Riaz Lakdawala, Shahryar Noordin

**Affiliations:** 1 Orthopaedic Oncology, University of Iowa, US; 2 The Indus Hospital Research Centre Karachi, PK; 3 Department of Medicine, Aga Khan University Karachi, PK; 4 Clinical Trials Unit, Aga Khan University Karachi, PK; 5 Department of Surgery, Medical College, Aga Khan University Karachi, PK; 6 Department of Surgery, Medical College, Aga Khan University Karachi, PK

**Keywords:** Knee arthroplasty, total knee replacement, skin closure, surgical site infection, prolene sutures, skin staple sutures

## Abstract

**Introduction:**

Knee arthroplasty also known as the total knee replacement is an orthopedic surgical procedure done to resurface the knee that has been severely damaged by arthritis. After the completion of the surgical procedure, the skin closure is done. The optimal goal of skin closure after the procedure is to promote rapid healing and an acceptable cosmetic result while minimizing the risk of infection. Skin closure after knee arthroplasty is done by using either of the two widely used sutures i.e., polypropylene (Prolene) sutures or the skin staple sutures. There are no standard guidelines as which type of the suture should be used. The present study aims to compare the incidence of surgical site infections (superficial and deep) for Prolene vs staple sutures in the bilateral knee arthroplasty patients within 6 weeks for superficial and within 90 days for deep infection.

**Methods:**

This study will be conducted as an open blinded, parallel design, equivalence randomized controlled trial. The patients would be selected and randomized in 1:1 ratio to receive either of the two interventions i.e., Prolene or Staples. Patients undergoing unilateral or staged total knee replacement (TKR) were excluded.

**Analysis:**

The normality assessment will be done using Shapiro Wilk test. Cox proportional hazard regression will be used to check the univariate and multi-variable associations of independent variables with the outcome. Both intention to treat analysis and per protocol analysis would be performed.

**Ethics and Dissemination:**

All the required approvals will be taken from the ethical review committee. Informed consent will be taken form the patient to enroll him/her in the study. Results of the study will be disseminated to the study participants, public health and clinical professionals and would also be published in a reputable international journal.

The trial is registered at *clinicaltrials.gov* and UIN of the registry is NCT04492852.

**Highlights:**

- Post-operative surgical site infections and complications are a major concern nowadays.
- Skin staples are not widely used as compared to Prolene because they are expensive and not easily available in every hospital.
- There are no standard guidelines as which type of the suture should be used.
- The type of sutures is being selected on the orders and wishes of the surgeon at the time of skin closure.

## 1. Introduction

With the increasing advancements in the science of accelerated rehabilitation, more pressure has been placed on the shoulders of the surgeons to perform more surgeries in a day and to reduce the length of hospital stay of the patients [1]. Knee arthroplasty is an orthopedic surgical procedure done to resurface the knee that has been severely damaged by arthritis [2]. It is a procedure performed to relieve the pain and minimize the damage and disability done to the knee [3]. Osteoarthritis is the most common cause resulting in the need for knee arthroplasty [4]. Osteoarthritis is the most common form of arthritis that affects millions of individuals worldwide [5]. Osteoarthritis is a degenerative disease of the joints that affects the joint cartilage and the bones surrounding it. It can affect almost any joint in the body but most commonly if affects the joints of knees, hands, spine and hip [5]. It causes pain, stiffness, grating sensation, loss of flexibility and bone spurs.

The damage and disability done to the knee by osteoarthritis is treated by doing knee arthroplasty in which parts made up of metal or plastic are used to cover the ends of the bones forming the knee joint [5]. After the completion of the surgical procedure, the skin closure is done. The optimal goal of skin closure is to promote rapid healing and an acceptable cosmetic result while minimizing the risk of infection [6]. Skin closure after knee arthroplasty is done by using either of the two widely used sutures i.e. polypropylene (Prolene) sutures or the staple sutures [7]. Prolene sutures are made up of a synthetic steroisomer known as polypropylene. It is a monofilament non-absorbable, sterile surgical suture [8]. They are indicated for use in general soft tissue. It provides permanent tensile strength retention in tissue, even in the presence of infection [8]. These sutures are exceptionally smooth for an easy passage through the tissue. Prolene sutures are widely used in cardiovascular, orthopedics, ophthalmic, and neurological surgical procedures [8]. Another type of sutures that are used to close the surgical wound are the staple sutures. They are used as an alternative to the traditional Prolene sutures [1]. They are non-absorbable and usually used on surgical wounds that are big, complex, or hard to close by using Prolene. These are specialized staples made up of titanium, stainless steel or plastic [9].

### 1.1 Rationale

Post-operative surgical site infections and complications are a major concern nowadays. It not only increases the length of hospital stay of the patient but, it also increases the healthcare cost and burden on the healthcare system. Both these types of sutures are commonly used to close the wounds after surgery. Skin staples are not widely used as compared to Prolene because they are expensive and not easily available in every hospital. A study of hip arthroplasty patients has shown that the risk of developing an infection in wounds closed with staples is 4 times as compared to the wounds closed with Prolene sutures [10]. A meta-analysis concluded that the risk of a wound infection was three times greater in wounds closed with staples as compared to the Prolene [6]. The results have been inconclusive of the studies conducted on the patients of knee arthroplasty [1].

In a study of 181 patients comparing staples and prolene, they reported significantly difference in complication rate between the two groups; the staples group had no complications while the suture group had 9 (9.1%) complications [11]. In another systematic review and metanalysis, they reported that both methods of closure are equivalent and suggested to choose for the economical method, sutures [12]. Moreover, there are no standard guidelines as which type of the suture should be used. The type of sutures is being selected on the orders and wishes of the surgeon at the time of skin closure.

### 1.2 Objectives

#### 1.2.1 Primary objective

➢ The primary objective of the study is to compare the incidence of surgical site infections (superficial and deep) for Prolene vs staple sutures in the bilateral simultaneous knee arthroplasty patients within 6 weeks for superficial and within 90 days for deep infection.

#### 1.2.2 Secondary Objective

1. The secondary objective of the study is to compare the incidence of postoperative complications bleeding, Hematoma, Seroma, pain) within 2 weeks.
2. To compare the pain scores at the time of removal of Prolene sutures and staples.

### 1.3 Hypothesis

#### 1.3.1 Null Hypothesis

There is a difference in the incidence of surgical site infection in bilateral simultaneous TKR patients with wound closure by Prolene vs staple.

#### 1.3.2 Alternative Hypothesis

There is no difference in the incidence of surgical site infection in bilateral simultaneous TKR patients with wound closure by Prolene vs staple.

## 2. Methods

### 2.1 Study Design

This study will be conducted as an open blinded, parallel design, equivalence randomized controlled trial. The patients would be randomized to receive either of the two interventions i.e., Prolene or Staples.

### 2.2 Study Site

The study would be conducted at the Aga Khan University Hospital, Karachi. The AKUH has a well-established medical records system and clinical trial unit (CTU) to facilitate clinical trials. Both the interventions being used in this study i.e. The patient would be recruited pre-operatively in the consulting clinic when the patient will book an appointment for the surgical procedure of bilateral simultaneous TKR. The intervention will be performed on the patient in the operating room while the knee arthroplasty surgery is being performed. Only one surgeon (surgeon A) will perform the intervention on the patient that will be selected prior to starting the study, to minimize the variability in incision techniques and post-operative management.

### 2.3 Study Interventions

The study will have 2 intervention arms. The patients will be randomized to receive either Prolene sutures for wound closure or staple sutures for wound closure. After the application of the intervention, the routine care would be given to the patients postoperatively.

### 2.4 Randomization

The patients would be randomized in 1:1 allocation to either receive Prolene sutures or the staple suture. Randomization will be done by the clinical trials unit (CTU) of AKUH using the specialized computer software. The computer-generated random allocation will ensure that nobody including the investigator, or the study team are able to influence it. Simple randomization process will be carried out using a block technique to assign the allocation. After completing the process of randomization, the details identifying the patient and the envelope number would be recorded on a form and will be returned to the trial administration to confirm the recruitment of the patient into the study.

### 2.5 Blinding

This study would be an open label trial. Due to the different nature of the visible marks of the sutures, it would not be possible to blind the study team, the PI, the surgeon, and the outcome assessor (SSI nurse) from the intervention administered. The surgical site infection nurse who assesses the surgical wounds post-operatively in the hospital as well as in clinics during follow up visits would be blinded to the hypothesis of the study. The other outcome assessors such as the pathologist who will conduct tests on the specimen taken from the surgical site, to confirm the presence of an infection would be blinded to the intervention.

### 2.6 Study Population

All bilateral simultaneous knee arthroplasty patients presenting at the outpatient clinics and opting to undergo surgery at AKUH Karachi, Pakistan.

### 2.7 Eligibility Criteria

#### 2.7.1 Inclusion Criteria

The study will include the adult patients of age 40–70 years [6], undergoing bilateral simultaneous knee arthroplasty at AKUH Karachi. Patients from both the genders would be included. Patients having American Society of Anesthesiologists (ASA) level of I, II and III pre-operatively would be included in the study. Patients having functional class of I, II and III pre-operatively would be included in the study. Only Patients opting for elective bilateral simultaneous knee arthroplasty would be included in the study, as emergency conditions such as trauma or fracture may complicate wound closure(3]. Only those patients would be included in the study who are opting to undergo bilateral simultaneous knee arthroplasty under care of the selected surgeon (A) for the study. Patients undergoing surgery should have the midline incision and through para-patellar approach used for making the incision will be included in the study.

#### 2.7.2 Exclusion Criteria

The Patients who are unwilling to consent and the patients unable to comprehend due to the language barrier will be excluded. Patients who have a Glasgow Coma Scale i.e. GCS<15 (cognitive impairment) will be excluded. Patients undergoing a knee revision surgery would be excluded from the study [16]. Patients having a previous incision/scar in the operative field will be excluded [1]. Patients having documented allergy to Prolene or stainless steel would be excluded [8]. Patients having a documented underlying malignancy will be excluded [3]. Patients undergoing unilateral TKR or staged bilateral simultaneous TKR would also be excluded from the study.

### 2.8 Sample Size

The sample size was calculated via open epi software version 3.01. The level of significance was kept at 5% with a power of 80%. The percentage of exposed (staple sutures) with outcome (surgical site infection) was 66%. The percentage of non-exposed (Prolene sutures) with outcome (surgical site infection) was 33%. The estimated risk ratio taken from a study was 2. After adding the non-response rate of 10% the final sample size came out to be 82 patients with 41 patients in each arm [3].

### 2.9 Recruitment

For this study, the non-probability consecutive sampling technique will be used. All the patients opting for surgeon A to undergo elective bilateral simultaneous TKR during the specified period for recruitment will be included in the study. By using the consecutive sampling technique, the probability to miss out on participants would be relatively small. Firstly, the patients opting to undergo bilateral simultaneous TKR under care of the selected surgeon would be identified and would be assessed against the eligibility criteria of the study. Secondly, the eligible patients would be briefed about the study and the interventions of the study. Furthermore, an informed consent would be obtained from the patient. The duration of the trial would be 2 years. During the first year of the study, the participants would be recruited until the desired sample size is achieved. After the surgical procedure, the patients will be followed for a year to assess the incidence of the surgical site infection.

### 2.10 Outcomes

#### 2.10.1 Primary Outcome

Primary outcome of the study is the incidence of surgical site infection (superficial or deep) within 6 weeks for superficial infections and 90 days for deep infections. SSI is defined as an infection that occurs after surgery in the part of the body where the surgery took place within 90 days of surgery [13]. Based on the literature, we anticipate infection rate in the range of 2%–7% [14,15].

#### 2.10.2 Secondary Outcome

The secondary outcomes of the study include the incidence of post-operative complication that includes seroma, hematoma, pain, bleeding within 2 weeks of the surgery. Another outcome of the study would be to assess and compare the pain scores at the time of suture removal.

### 2.11 Outcome Assessment

The outcome will be assessed by the surgical site infection nurse. They will assess the incidence of infection by clinical examination of the wound and pre-validated tools that they are already using during the post-operative follow ups. The secondary outcomes will be assessed by the surgery team through clinical examination. The secondary outcome of pain would be assessed by using the verbal pain score assessment. A case report form (CRF) would be maintained for everyone participating in the study. All the information regarding the individuals i.e., baseline characteristics, lab reports, scans, forms filled by SSI nurses would be maintained in CRF.

## 3. Plan of Analysis

### 3.1 Descriptive statistics

STATA version 15.0 will be used for the statistical analysis. Initially the descriptive statistics will be calculated. For the quantitative variables, the normality assumption would be checked using the Shapiro Wilk test. The mean and standard deviation will be calculated and reported. Median and interquartile range will be reported if the normality assumption for that variable is violated. The qualitative variables will be expressed as the frequencies and proportions.

### 3.2 Univariate and Multivariable Analysis

The Cox Proportional Hazard regression will be used for the univariate and multivariable analysis. Univariate analysis will be conducted, and crude risk ratios and their 95% confidence intervals will be obtained. All significant independent variables at the univariate stage will be regressed by using cox proportional hazard regression using the stepwise method in the multivariable model and the adjusted risk ratios will be obtained. Both per-protocol analysis and intention to treat analysis (ITT) would be performed.

## 4. Ethical Considerations

### 4.1 Harms and Adverse Events

There are no anticipated harms in the study. In case of any unanticipated adverse event, the Ethical review committee (ERC) of AKUH would be informed and the recommended actions would be taken.

### 4.2 Ethical Review Committee

This protocol is exempted from Ethical review committee as it’s just a protocol. All the required approvals will be taken from the ERC, CTU and from the Musculoskeletal and sports medicine service line chief. The trial is registered at *clinicaltrials.gov* and UIN of the registry is NCT04492852.

### 4.3 Informed Consent

Informed consent will be taken form the patient to enroll him/her in the study. All the required information regarding the study will be given to the patient during the process of taking informed consent. A copy of the consent form will be provided to the patients. The patients will be explained about benefits and risks attached to the study.

## 5. Dissemination of the Study Findings

Results of the study will be disseminated to the study participants, public health, and clinical professionals. The results obtained from the trial would be disseminated to all the surgeons of service line to ensure a standard practice. The results would also be published in a reputable international journal.

## Additional File

The additional file for this article can be found as follows:

10.29337/ijsp.153.s1 The additional file includes the CONSORT checklist of reporting randomized trials. CONSORT 2010 checklist of information to include when reporting a randomised trial*.
